# Integrative Comparative Assessment of Cold Acclimation in Evergreen and Deciduous Iris Species

**DOI:** 10.3390/antiox11050977

**Published:** 2022-05-16

**Authors:** Lingmei Shao, Tong Xu, Xiaobin Wang, Runlong Zhang, Xiuyun Wang, Ziming Ren, Jiaping Zhang, Yiping Xia, Danqing Li

**Affiliations:** Genomics and Genetic Engineering Laboratory of Ornamental Plants, College of Agriculture and Biotechnology, Zhejiang University, Hangzhou 310058, China; 12116125@zju.edu.cn (L.S.); 22016252@zju.edu.cn (T.X.); xiaobinwang@zju.edu.cn (X.W.); 21916201@zju.edu.cn (R.Z.); xiuyunwang@zju.edu.cn (X.W.); zimingren@zju.edu.cn (Z.R.); 0015604@zju.edu.cn (J.Z.)

**Keywords:** leaf freezing tolerance, leaf microstructure, sugar, phytohormone, ROS scavenging, proline, gene expression, deacclimation

## Abstract

Cold acclimation (CA) is a strategy which plants have evolved to increase freezing tolerance. Global climate change could obstruct CA and raise the probability of winter injury, especially for evergreens. Hence, understanding the regulatory mechanism of CA is crucial to improve freezing tolerance in evergreen plants. A comparative study on a pair of closely related evergreen and deciduous iris species in response to cold through CA was conducive to uncovering and complementing the knowledge of CA. We investigated morphological, physiological and biochemical changes, as well as the expression of associated genes in the functional leaves of both iris species from natural CA to deacclimation. Briefly, fast and strong CA in the evergreen iris might cause early expressions of *BAM1*, *NCED3*, *GPX6*, etc., which leads to strong enzyme activity of starch degradation, abscisic acid biosynthesis and reactive oxygen species scavenging. Additionally, genes belonging to the antioxidant system were mainly induced during deacclimation. These results suggest that interspecies differences in the leaf freezing tolerance of irises are associated with the rate and degree of CA, which activates multiple signaling networks with complex interactions and induces the transcription of cold-responsive genes. Moreover, the ICE–CBF–COR signaling cascade may integrate and initiate diverse cold-responsive pathways during CA of the evergreen iris. The findings of this study provide valuable insight to further research on CA mechanisms and implicate genes which could support breeding strategies in herbaceous perennials under climate changes.

## 1. Introduction

Low winter temperatures are determinants that force restrictions on the geographic distribution and development of plants [1]. To adapt to low temperatures in mid-winter, plants develop freezing tolerance when exposed to low, nonfreezing temperatures [2]. This ingenious mechanism is known as cold acclimation (CA), which activates various physiological responses, such as the accumulation of osmotic substances, antioxidants and phytohormonal homeostasis [3,4]. CA can trigger growth cessation and dormancy and protect plant cells from cold-induced damage through the activation of *cold-regulated gene*s (*COR*s) [2]. Recent studies have shown that global climate change greatly impedes the development of the CA process, making perennials more likely to sustain freeze-related injuries [5,6]. To date, most of the knowledge of plant CA is based on studies of *Arabidopsis* and other deciduous model plants, which is lacking in evergreen herbs. Unlike annuals or deciduous perennials, which survive in winter in the form of seeds or underground organs buried deep in the soil, the leaves of evergreen herbaceous perennials directly exposed to the air are more vulnerable to freezing damage. Thus, understanding the molecular mechanism of the CA process is essential for improving leaf freezing tolerance (LFT) and maintaining high aesthetic value in evergreen herbaceous perennials.

Transcription factors cascade which in response to freezing stress during the process of CA have been studied intensively. It could regulate the expression of *COR*s encoding cryoprotective proteins which protects plant cells against cold-induced damage [2]. Of these reported cascades, the ICE–CBF/DREB–COR pathway plays a central role during CA [7,8]. C-repeat binding factors (CBFs) might integrate stress-responsive substrates; subsequently, sugars and phytohormones interact and synergistically facilitate improvements in freezing tolerance in plants by directly or indirectly participating in the ICE–CBF–COR cascade [9,10]. Accumulating evidence has revealed that the degradation of starch and accumulation of soluble sugars, especially sucrose, help to enhance freezing tolerance during CA [11]. Genes encoding beta-amylase (BAM, a key enzyme in starch degradation) have been identified as CBF regulons and work during CA by regulating sugar concentrations [12]. In addition, studies have suggested that sugars acting as signaling molecules can trigger abscisic acid (ABA) and other stress-responsive factors (such as antioxidant reaction) to withstand cold conditions [13].

Phytohormonal regulation in the cold stress response is a complex but precise process involving the crosstalk of multiple endogenous phytohormones [14]. The application of exogenous ABA activates the cold stress response and elevates the freezing tolerance of plants during CA [15,16]. Notably, a significant elevation of freezing tolerance observed in the overexpression of *auxin-upregulated 3.2* (*GH3.2*, a negative component in auxin signaling) in *Oryza sativa*, which leads to the alleviation of oxidative damage and membrane injury [17]. Additionally, cold-induced jasmonic acid (JA) signaling may function synergistically with ABA signaling via jasmonate-insensitive 1 (MYC2) and PYR1-LIKE 6 (PYL6) protein to regulate the CBF pathway [18,19], which results in alleviating chilling injury [20]. Previous studies showed the increase in reactive oxygen species (ROS) in plants is the inevitable result of cold stress [21], and the overaccumulation of ROS in plant cells is extremely toxic and eventually causes cellular death [22]. Indeed, ROS scavenging mechanisms occur throughout various physiological responses during CA. Sugars, ABA and proline act as antioxidants, protecting plants from injuries caused by overaccumulated ROS in cold stress responses [23,24,25].

The intensity and rate of the cold stress response have a significant effect on overwintering plants because cold stress affects the survival and quality of revegetation and flowering in overwintering plants [26]. Rhizomatous irises are one of the most popular herbaceous perennials for gardens worldwide due to their brilliant ornamental traits and tens of thousands of attractive cultivars. Our previous studies found that both evergreen and deciduous irises adapted well to the climates of eastern China, where subzero temperatures occur in the winter [27]. In this study, we focused on the differences in evergreen and deciduous plants in response to cold through CA. On the premise for the lack of corresponding mutants, we selected a pair of closely related irises as representatives for a comparative study. The selected *Iris hexagona* (evergreen) and *I. pseudacorus* (deciduous) belong to the sect. *Limniris* [28,29] and are both hydrophytes with similar leaf shapes.

In this study, we compared the freezing tolerance of functional leaves (at the status of active growth), which avoids confusion caused by their different winter dormancy characteristics and foliar habits. Our study aimed to clarify the roles of sugars, phytohormone signaling and ROS scavenging in acquiring LFT during CA in two species under contrasting durations of low-temperature exposure. In this study, sugar, phytohormone and antioxidant metabolism were associated with freezing tolerance in iris plants, which agreed with our previous study [30]. Notably, the early and rapid accumulation and the interactions of soluble sugar, ABA and superoxide dismutase (SOD) activity in the leaves of evergreen irises were determinants to improve LFT and protect plants from freezing during CA. In addition, the ICE–CBF–COR pathway might integrate sugars, ABA, antioxidants and other cold-responsive substances. Overall, we proposed a putative mechanism in iris plants during CA, which could assist in the success of elevating the LFT of evergreen herbaceous perennials using molecular breeding under global climate change.

## 2. Materials and Methods

### 2.1. Plant Materials and Treatments

**Figure 1:**
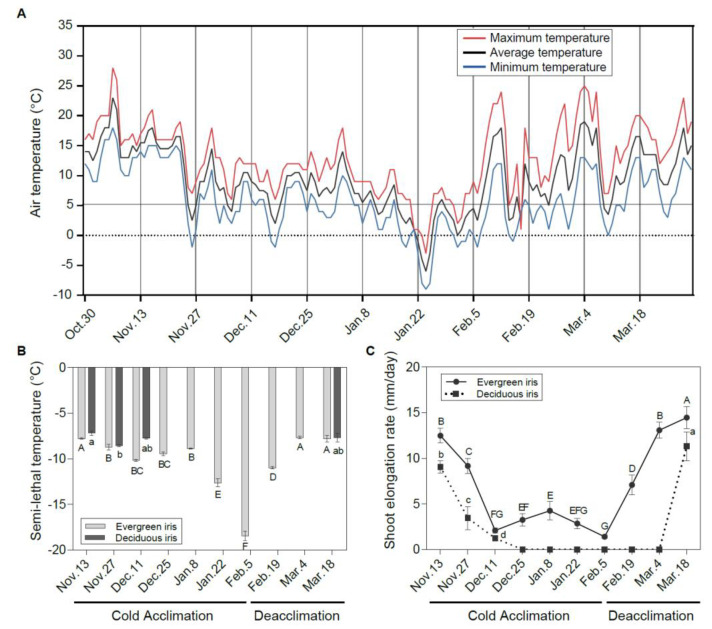
Freezing tolerance and vegetative growth status of leaves in evergreen and deciduous irises under natural cold acclimation and deacclimation. (**A**) Changes in air temperature from 30 October to 21 March 2021 in the field. The maximum temperature, average temperature and minimum temperature are indicated by red, black and blue lines, respectively. (**B**) Changes in semi-lethal temperature (LT_50_) for functional leaves in evergreen and deciduous irises. (**C**) Alterations in the shoot elongation rate in evergreen and deciduous irises. Cold acclimation process, sampling from 13 November to 5 February 2021; deacclimation process, sampling from 19 February to 18 March 2021. The data are the means of three biological replicates, with different letters indicating significant differences between treatments according to Duncan’s multiple range test at *p* < 0.05. No functional leaves could be sampled from 25 December to 4 March 2021 in deciduous irises. Capital and lowercase letters represent significant differences for relevant parameters within evergreen and deciduous irises (*p* < 0.05), respectively.

In this experiment, two-year-old *I. hexagona* ‘Bryce Leigh’ and *I. pseudacorus* propagated by division were obtained from a local company (Hangzhou Landscaping Incorporated, China) and planted in the Resources Nursery for Flower Bulbs and Herbaceous Perennials at Zhejiang University, Hangzhou (29° 11′–30° 33′ N, 118° 21′–120° 30′ E), China. Winter usually begins in late November in Hangzhou and lasts until mid-March of the following year. The average winter temperatures in Hangzhou are 5.8~7.4 °C, and the annual minimum temperature usually occurs from late January to early February 2021 [31]. In our study, the daily air temperatures were measured in the experimental field from 13 November to 18 March 2021 and are depicted in Figure 1A. *I. hexagona* and *I. pseudacorus* are described as evergreen and deciduous irises here, respectively, to promote understanding. Two uniformly sized iris species were planted in pots (one plant per pot), which were filled with a 3:1 (*v*/*v*) mixture of light sandy loam soil and peat. Pots were placed in the field for a natural overwintering experiment. For the convenience of sampling, the 120 pots of each species were randomly and equally arranged as 3 sample plots. Each sample plot indicated a replication; thus, each treatment contained three replications. In accordance with the methods of Li et al. [27], routine management and fertilization of iris plants were conducted. An intelligent temperature and humidity recorder (Zeda Instruments, ZDR-F20, Hangzhou, China) was placed in the experimental site to record the temperature changes during the experiment.

In our previous study [27], we measured the freezing tolerance of two iris species (*I. hexagona* and *I. pseudacorus*) from two cycles of 2014–2015, and 2015–2016. These results confirmed that the differences in freezing tolerance between two iris species were stable though the field conditions. Thus, in this study, samplings and measurements were taken every two weeks at ten stages ranging from 13 November to 18 March 2021. On each sampling day, whole functional leaves of both iris species were taken. The third and fourth actively growing leaves from the central part to both sides in a plant were considered as functional leaves [27,30]. Partial fresh samples were used for the determination of semi-lethal temperature (LT_50_) and microstructure observations, and the others were chopped and divided into 0.30 g per portion and then rapidly frozen in liquid nitrogen and immediately stored at −80 °C until further analyses.

### 2.2. Evaluation of Leaf Freezing Tolerance

Leaf LT_50_ is a widely recognized index to evaluate the freezing tolerance of plants. According to the method of Xu and Chen [32], the measured relative electrolyte conductivity (REC) value was conducted for logistic nonlinear fitting in Origin Pro (Origin Lab Corp, v9.1, Northampton, MA, USA) to obtain the leaf LT_50_ of each iris sample. Specifically, samples from each sampling were exposed to a temperature-controlled freeze–thaw cycle, which included an ice-nucleation process of all samples and a stepwise reduction in temperatures using an ethanol cooling bath (Hanuo instruments Co. Ltd., HX-3030, Shanghai, China). The target temperatures were set as presented in Table S1. Samples of both iris species for LT_50_ determination were equally separated into six portions. After every 1.5 h, one portion was removed from the target temperature and thawed at 4 °C for 6 h. Leaf tissues exposed to 4 °C served as control (unfrozen) samples. After thawing, samples (including controls) were immersed in 25 mL ultrapure water and vacuum-infiltrated for 14 h. The electrolyte leakage (EL) of thawed leaves was measured with a digital conductivity meter (Ohaus Instruments, DDS-12A, Shanghai, China); then, samples were incubated in a boiled water bath for 20 min to determine the maximum EL. The REC was calculated as follows [30,33]:

REC (%) = [EL_thawed leaves_/EL_maximum_

− Average (EL_control leaves_/EL_maximum_)]/

[100 − Average (EL_control leaves_/EL_maximum_)] × 100

In addition, the overwintering processes of the two irises were divided into different periods according to the changes in their LFT.

### 2.3. Morphological Measurements and Growth Indices

To determine the shoot elongation rate (SER), we measured changes in the shoot height at ten sampling stages. The aerial portions of the two species were clipped at a height of 10.00 cm above the soil surface two weeks before each sampling to clearly measure plant growth. The calculation formula of SER is as follows:

SER = (L_x_ − L_y_)/T

where L_x_ (mm) is the maximum shoot length of each sample on a certain sampling date x, L_y_ (mm) is the clipped shoot length on sampling date y (Ly = 100 mm in this study), x is the adjacent sampling stage after y, and T (d) is the days elapsed between x and y (T = 14 days in this study).

The relative chlorophyll content (RCC), maximum leaf width (LW), fresh weight (FW) of functional leaves and number of functional leaves (NFL) of ten representative plants in three plots were randomly measured every two weeks. The RCC was measured with a chlorophyll meter (Konica Minolta Sensing, Inc., SPAD-502 PLUS, Tokyo, Japan). 

### 2.4. Observation of Leaf Microstructures after Exposure to Low Temperatures

To detect leaf microstructures of two iris species after exposure to low temperatures, functional leaves of each species without diseases or pests were randomly selected for histological observation on 27 November 2021. In brief, leaves 5.00–6.00 cm away from the tips were vertically cut into small segments with a width of 0.50 cm. On the one hand, after fixation with formalin–acetic acid–alcohol (FAA) for 24 h, sections were made into paraffin slices according to the method of Yang [34] and stained with Safranin O-Fast Green. On the other hand, leaf epidermis slices were prepared following the method of Sun and Jiang [35]. The small segments were immersed in a 1:1 (*v*/*v*) mixture of H_2_O_2_ and CH_3_COOH for 5 h, and the upper and lower epidermis were separated from the mesophyll cells. The processed leaves were made into slices. Ten fields of vision were randomly selected for each sample, and the microstructures of selected leaves were observed with an optical microscope (Olympus, CPH-500Z, Tokyo, Japan).

### 2.5. Determination of Carbohydrate Concentrations

The concentrations of total soluble sugar (TSS), sucrose and starch were determined by the anthrone method, according to Wang et al. [36]. Approximately 0.30 g of iris leaf tissue was first mixed with some arenaceous quartz and 5 mL of 80% ethanol and then ground. The homogenates were transferred to 10 mL centrifuge tubes and centrifuged at 4000 × g for 5 min (repeated three times). The supernatant was collected, then distilled water was added to the supernatant at a constant volume of 50 mL and the extraction was repeated three times. TSS and sucrose concentrations were measured following the method of Wu et al. [37]. Then, the residues left in the centrifuge tubes were dried at 80 °C for starch extraction using 52% HClO_4_, as described by Wang et al. [38].

### 2.6. Measurement of Leaf Phytohormone Levels

The measurements of plant phytohormones were performed by enzyme-linked immunosorbent assay (ELISA), as described previously by Yang et al. [39]. Briefly, 1 g of frozen samples was homogenized and extracted with 2 mL precooled 80% (*v*/*v*) methanol extraction medium containing 1 mM butylated hydroxytoluene as an antioxidant. The extracts were centrifuged at 5000× *g* for 10 min (4 °C) after incubation at 4 °C for 4 h. The supernatants were purified using an SPE-C18 column (SepPak-C18, Waters Corp, Milford, MA, USA). Specifically, the SPE-C18 column was prewashed with 80% methanol for 1 min before purification and sequentially washed with 5 mL of 100% methanol, 5 mL of 100% diethyl ether and 5 mL of 100% methanol in turn after purification. The SPE-C18 columns could be recycled approximately 10–15 times. The phytohormone fractions were dried under N_2_ and dissolved in sample diluent containing 2 mL of phosphate-buffered saline (PBS, pH 7.5), 0.1% (*v*/*v*) Tween-20 and 0.1% (*w*/*v*) gelatin (pH 7.8) for analysis by ELISA.

The standards for IAA, ABA, JA, and GA_3_ (Yuanye Biochemical Company, Shanghai, China) were used to quantify the levels. The mouse monoclonal antigen, antibodies against IAA, ABA, JA, or GA_3_, and immunoglobulin G-horseradish peroxidase (IgG-HRP) used in the ELISA were produced at the Phytohormone Research Institute, Beijing, China. The method for the quantification of ABA, JA, GA_3_, and IAA by ELISA was described by Yang et al. [39].

### 2.7. Measurements of MDA, Proline, SP Contents and SOD Activities in Leaf Tissues

Frozen functional leaf samples of two iris species were used to determine these physiological and biochemical parameters. The measurement and calculation of malondialdehyde (MDA) were based on the thiobarbituric acid (TBA) reaction [40]. The proline content was determined as described by Bates et al. [41], and the soluble protein (SP) content was measured using the Coomassie blue staining method [42]. Additionally, SOD activity was measured using the nitroblue tetrazolium (NBT) reaction [43], with a slight modification.

### 2.8. Expression Analysis of Genes Involved in Crucial Pathways during Natural Cold Acclimation and Deacclimation

To explore the expression patterns of genes related to the above indices crucial to natural CA and deacclimation, total RNA was extracted from the functional leaf tissues of two iris species sampled on 13 November, 27 November, 11 December, 5 February and 18 March 2021 with a total RNA extraction kit (Tiangen, Beijing, China). The purity and concentration of RNA were assessed with a NanoDrop (ND-1000) spectrophotometer (Isogen Life Science, Utrecht, Netherlands). First strand cDNA was synthesized with the PrimeScript RT Reagent Kit with a gDNA eraser (TaKaRa, Kyoto, Japan).

Candidate genes related to plant freezing tolerance (listed in Table S2) were selected from the literature. Quantitative real-time PCR (qRT–PCR) was performed to determine the gene expression of these genes using the primers in Table S2. Primers were designed according to the iris transcriptome from our laboratory [44] and verified in two iris species by clone sequencing, with an expected product fragment size of 100–200 bp and similar Tm (melting temperature, Tm) value (about 60 °C) for each primer. The *β-Actin* gene was used as an internal reference for the normalization of qRT–PCR [45], and relative gene expression levels in each sample were calculated using the standard 2^−ΔΔCt^ algorithm with three biological replicates [46]. qRT–PCR was conducted with TB Green^®^ Premix Ex Taq (TaKaRa, Kyoto, Japan) on a CFX ConnectTM Real-Time PCR Detection system (Bio-Rad, Hercules, CA, USA). Every reaction contained 4 μL of cDNA, 5 μL of TB Green, 0.5 μL of forward primer and 0.5 μL of reverse primer. The qRT–PCR cycle parameters were as follows: 2 min at 95 °C; 39 cycles of 5 s at 95 °C and 30 s at 55 °C; and a melting curve program of 5 s at 95 °C, 5 s at 65 °C and 5 s at 95 °C.

### 2.9. Statistical Analyses

Statistical analyses were performed using SPSS 23 (IBM Corporation, Armonk, NY, USA), and figures were generated in GraphPad Prism 6 (San Diego, CA, USA). All data were measured from at least three biological replicates. One-way ANOVA and Duncan’s multiple repeat comparative analysis were performed on the experimental data to compare the differences among various samples at a significance level of 0.05. Correlation analyses between LT_50_ and physiological data were performed using Pearson’s two-tailed tests.

## 3. Results

### 3.1. Changes in Leaf Freezing Tolerance of Two Iris Species under Natural Cold Acclimation and Deacclimation

With the decrease in air temperature (Figure 1A), irises initiated the CA process. The LFT of leaves represented by LT_50_ changed in different ways in the two species under natural CA and deacclimation (Figure 1B). Generally, the LFT of the evergreen iris presented small changes during early CA before 8 January 2021. After long-term natural CA and exposure to extremely low temperatures, it increased prominently and reached a maximum on 5 February 2021. Subsequently, the evergreen iris quickly completed deacclimation and lost LFT with rising temperatures (from 5 February to 18 March 2021). However, the LFT of the deciduous iris was only significantly enhanced from 13 to 27 November 2021 during CA, but subsequently decreased slightly. The LT_50_ values in leaves of deciduous irises were coincident with those of the evergreen irises, except for on 11 December 2021. According to the changes in LFT in the evergreen iris and the alterations in air temperature, the overwintering cold responses of two irises were divided into two processes—CA and deacclimation—and 5 February 2021 was set as the boundary.

### 3.2. Changes in Vegetative Growth Status of Two Iris Species during Overwintering

Before 11 December 2021 (the early stage of CA), the SER decreased consecutively in both species (Figure 1C). The deciduous iris ceased growth after 25 December 2021, whereas the SER of the evergreen iris was detected above 1.40 mm/day throughout the experiment. In early March 2021 during deacclimation, the SER of the evergreen iris had already increased to the level observed on 13 November 2021 before CA, whereas the SER in the deciduous iris was just beginning to increase. In short, the evergreen iris grew through the cold winter, whereas the deciduous iris suspended growth in mid-winter. Other vegetative growth indices, such as RCC and LW, were also determined in both iris species during CA and deacclimation (Table S3).

Considering the correlation between LFT and vegetative growth indices, significant correlations between LT_50_ and the SER value and FW were only observed in evergreen irises (Table 1). Furthermore, the SER was considered the most important vegetative growth index and exhibited a significantly high correlation with LW and FW in the evergreen iris and a high correlation with RCC and FW in the deciduous iris.

### 3.3. Leaf Microstructure Comparisons between Two Iris Species after Exposure to Low Temperatures

**Figure 2:**
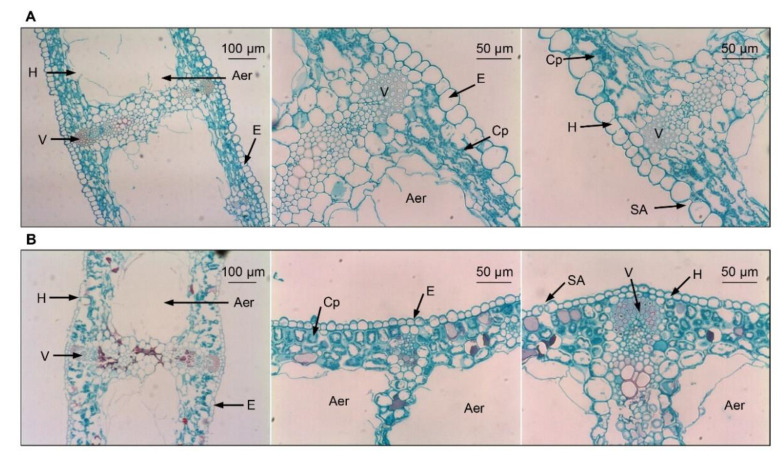
Leaf microstructures of evergreen and deciduous irises after exposure to low temperatures (during cold acclimation on 27 November 2021). (**A**) Leaf microstructures of evergreen irises. (**B**) Leaf microstructures of deciduous irises. Aer, aerenchyma; Cp, chloroplast; E, epidermis; H, hypodermis; SA, stomatal apparatus; V, vascular bundle.

Leaf microstructures of both iris species are similar, with notable cavities in the middle of leaves and undifferentiated spongy and palisade parenchyma in mesophyll cells (Figure 2A,B). Specifically, the leaves of the evergreen iris are thicker, with larger foliar epidermal cells compared with the leaves of deciduous iris (Table S4). In contrast, the stomatal apparatus distribution of the deciduous iris was slightly denser than that of the evergreen iris due to the smaller stomatal apparatus size (Figure S1). 

### 3.4. Alterations in Carbohydrate Concentrations and the Expression of Related Genes in Leaf Tissues

**Figure 3:**
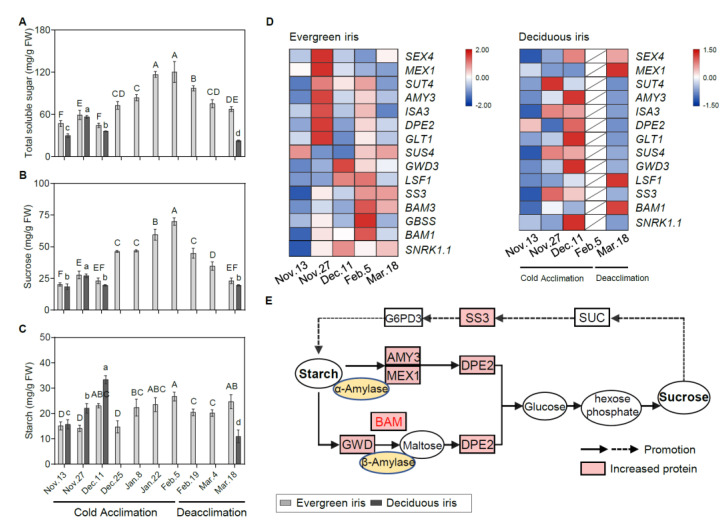
Physiological and molecular analyses of carbohydrates in the functional leaves of evergreen and deciduous irises under natural cold acclimation and deacclimation. (**A–C**) Changes in carbohydrate concentrations for functional leaves in evergreen and deciduous irises. For deciduous irises, no functional leaves could be sampled from 25 December to 4 March 2021. Cold acclimation process, sampling from 13 November to 5 February 2021; deacclimation process, sampling from 19 February to 18 March 2021. The data are the means of three biological replicates, with different letters indicating significant differences between treatments according to Duncan’s multiple range test at *p* < 0.05. The push pin represents standard error. Capital and lowercase letters represent significant differences for relevant parameters within evergreen and deciduous irises (*p* < 0.05), respectively. (**D**) Expression levels of sugar-metabolism-related genes expressed in evergreen and deciduous irises. Different colors indicate different levels of gene expression by quantitative real-time PCR; the data in each row were normalized and compared separately; blue indicates downregulation, and red indicates upregulation. Gene names are shown in Table S2. (**E**) A schematic illustration of sugar metabolism in evergreen and deciduous irises in this study.

Similar changing trends in TSS and sucrose concentrations were detected in both iris species during CA and deacclimation (Figure 3A,B). Notably, the TSS and sucrose concentrations in evergreen irises were markedly augmented as air temperature decreased and natural CA persisted and reached their maximum values of 120.45 mg/g FW and 69.96 mg/g FW on 5 February 2021, respectively, and then rapidly reduced during deacclimation. In addition, TSS and sucrose were highly correlated with LT_50_ in the evergreen iris (Table 1). Although the starch concentrations in the evergreen iris fluctuated, they gradually increased during CA and decreased during deacclimation (Figure 3C). As for deciduous iris, starch was rapidly synthesized during CA, accumulating twice during early CA (from 13 November to 11 December 2021) and reaching a maximum of 33.32 mg/g FW on 11 December 2021. However, a minimum of 10.99 mg/g FW was obtained after the deciduous iris went through deacclimation on 18 March 2021. 

To gain further insight into the molecular regulation of carbohydrates during CA and deacclimation, we investigated the gene expression involved in carbohydrate catabolism pathways. Overall, genes involved in the starch catabolism of the evergreen iris were mainly upregulated on either 27 November or 5 February 2021 (during early CA and when CA was completed), which was earlier than those in the deciduous iris (Figure 3D). For example, in the evergreen iris, one of the upstream genes, *alpha-amylase-like 3* (*AMY3*), in the starch catabolic process was considerably elevated 4.5-fold on 27 November 2021 in comparison with the first sampling date. In contrast, the expression of *AMY3* presented a remarkable induction in the deciduous iris on 11 December 2021, during early CA. Additionally, we observed that *BAM1/3* of the evergreen iris was dramatically upregulated during CA and exhibited a peak on 5 February 2021. However, *BAM1* of the deciduous iris was mainly upregulated on 18 March 2021, during deacclimation. The main upregulated genes are shown in a schematic illustration of sugar metabolism (Figure 3E).

For genes in the starch synthesis pathway, the upregulation of these genes in the evergreen iris occurred later than that in the deciduous iris. Specifically, a prominent increase in *starch synthesis* (*SS3)* was exhibited in the evergreen iris during deacclimation (on 5 February and 18 March 2021). Moreover, during CA, *SS3* of the deciduous iris was greatly induced sixfold on 27 November 2021 and fourfold on 11 December 2021, as compared with the first sampling date (Figure 3E). Notably, the expression of starch biosynthetic genes on 27 November 2021 was higher than that on 11 December 2021 in both species, whereas the starch concentrations on 11 December 2021 were lower than those on 27 November 2021, which was presumably due to the immediate degradation of starch and its transformation into other sugars to withstand cold on 27 November 2021, with the temperatures markedly dropping.

### 3.5. Effects of Cold Acclimation and Deacclimation on Phytohormone Levels in Leaf Tissues

**Figure 4:**
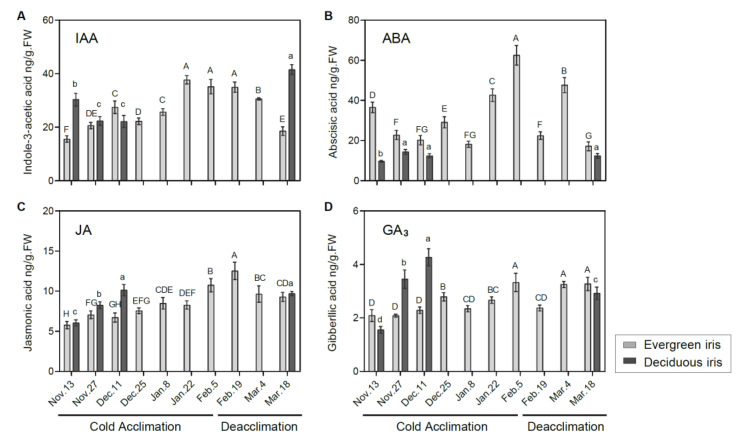
Measurements of endogenous phytohormones in the functional leaves of evergreen and deciduous irises under natural cold acclimation and deacclimation. Levels of (**A**) IAA, (**B**) ABA, (**C**) JA and (**D**) GA_3_. Cold acclimation process, sampling from 13 November to 5 February 2021; deacclimation process, sampling from 19 February to 18 March 2021. Values are the means of three biological replicates, and bars represent the standard error. Different letters indicate significant differences according to Duncan’s multiple range test at *p* < 0.05. Capital and lowercase letters represent significant differences for relevant parameters within evergreen and deciduous irises (*p* < 0.05), respectively.

The levels of IAA showed completely opposite changing trends in both iris species during the overwintering period. In the evergreen iris, the IAA level presented an overall upward trend, increasing by 125.74% from 13 November to 5 February 2021 during CA (Figure 4A), and decreased during deacclimation. Conversely, the IAA level of the deciduous iris gradually diminished before 11 December 2021 during CA, and a high level appeared on 18 March 2021 during deacclimation. Furthermore, we found that IAA levels were strongly correlated with LT_50_ values in evergreen irises (Table 1), indicating its importance in CA. Cold stress induces ABA accumulation in plants, which promotes the plants’ stress responses [47]. In our experiment, ABA levels of two iris species were highly correlated with their LT_50_ values (Table 1). In the evergreen iris, the ABA level presented an increasing trend from 11 December to 5 February 2021, and the maximum 62.54 ng/g FW occurred on 5 February 2021, whereas a gradual decline was observed during deacclimation and exhibited a minimum of 17.14 ng/g FW on 18 March 2021 (Figure 4B). However, the ABA level of the deciduous iris barely increased throughout the experiment. 

JA helps plants alleviate chill-induced injury [18]. Overall, the JA levels of both species exhibited a similar trend during the experiment, which gradually increased during CA and presented the opposite change during deacclimation (Figure 4C). Specifically, the JA level of the evergreen iris significantly increased during CA, and decreased markedly in the last two periods of deacclimation. In the evergreen iris, the level of GA_3_ presented an increasing tendency during CA and was maintained at a relatively high level during deacclimation (Figure 4D). Moreover, the GA_3_ level of the deciduous iris was continuously augmented during CA and significantly increased by 175.48% from 13 November to 18 December 2021.

### 3.6. Expression Changes in Genes Associated with Phytohormone Biosynthesis and Signal Transduction in Leaf Tissues

**Figure 5:**
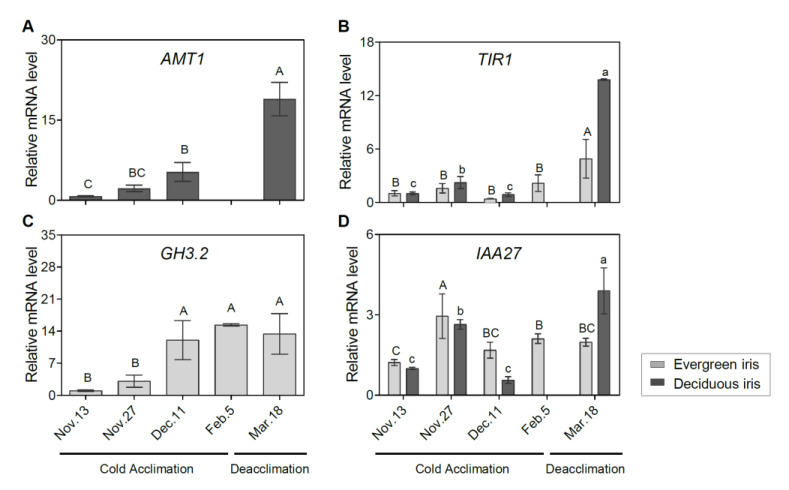
Expression changes of auxin-related genes in functional leaves of evergreen and/or deciduous irises under natural cold acclimation and deacclimation. (**A**) Expression levels of *AMT1* for deciduous iris. (**B**) Expression levels of *TIR1* for two iris species. (**C**) Expression levels of *GH3.2* for evergreen iris. (**D**) Expression levels of *IAA27* for two iris species. Cold acclimation process, sampling from 13 November to 5 February 2021; deacclimation process, sampling from 19 February to 18 March 2021. Values are the means of three biological replicates, and bars represent standard error. Different letters indicate significant differences according to Duncan’s multiple range test at *p* < 0.05. Capital and lowercase letters represent significant differences for relevant parameters within evergreen and deciduous irises (*p* < 0.05), respectively. Gene names are shown in Table S2.

To assess the molecular regulation of the phytohormone signaling pathway during iris overwintering, we determined the expression of related genes. The auxin biosynthetic gene *a-methyl tryptophan-resistant 1* (*AMT1*) was upregulated during CA and deacclimation in the deciduous iris (Figure 5A). Additionally, the expression of *transport inhibitor response 1* (*TIR1*) peaked on 18 March 2021 during deacclimation in both iris species (Figure 5B). Additionally, the expression of *GH3.2* was induced during CA and decreased during deacclimation in the evergreen iris (Figure 5C), which presented a similar trend to *indole-3-acetic acid inducible 27* (*IAA27*) (Figure 5D). 

**Figure 6:**
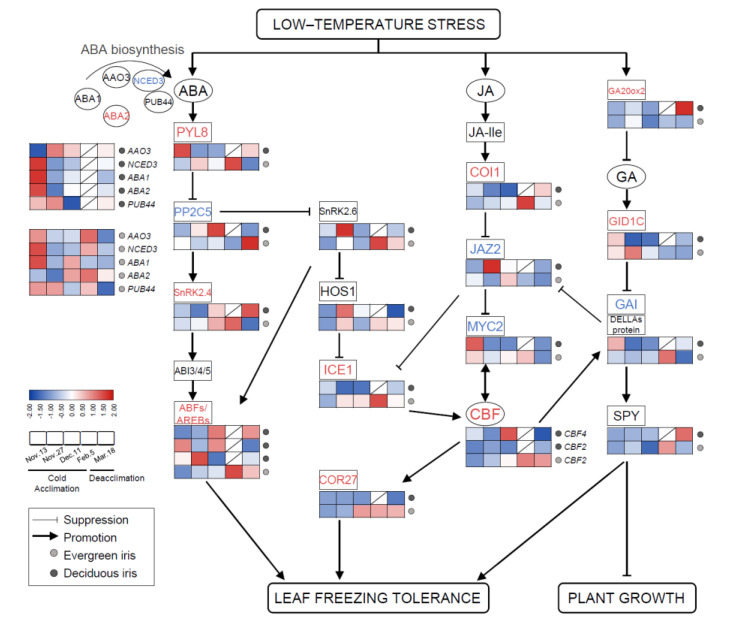
The involvement of plant phytohormone signaling in responses to cold stress and the expression levels of related genes in functional leaves of evergreen and deciduous irises. *CBF*s can be directly modulated by key components of JA signaling pathways. ABA and GA signaling are thought to facilitate improvements in freezing tolerance in irises, mainly by participating in the ICE–CBF–COR cascade. Arrows indicate promotion, whereas lines ending with a bar suggest suppression. Cold acclimation process, sampling from 13 November to 5 February 2021; deacclimation process, sampling from 19 February to 18 March 2021. Gene names are presented in Table S2.

In this study, the high expression of ABA biosynthesis genes was mainly observed on 13 November 2021 (at the beginning of CA) in both species (Figure 6); moreover, most of these genes were also significantly upregulated on 5 February 2021 (when CA was completed) in evergreen irises, such as *nine-epoxycarotenoid dioxygenase 3* (*NCED3*). After exposure to extremely low temperatures, the accumulation of ABA resulted in the upregulation of ABA signaling genes (positive regulation) on 5 February 2021 in the evergreen iris. In contrast, ABA signal transduction genes in the deciduous iris were mainly upregulated on 11 December 2021 (before leaf withering), during CA. For instance, the expression of the ABA receptor protein gene *PYR8* in the evergreen iris was elevated approximately twofold from 13 November to 11 December 2021 and fourfold from 13 November to 5 February 2021 during CA. However, *PYR8* of the deciduous iris only showed high expression on 13 November 2021 during CA. Notably, the expression of most ABA signal transduction genes in two irises exhibited opposite trends during deacclimation. 

Similar trends in JA signal transduction genes were found in both species during overwintering. In short, JA signaling genes in the deciduous iris were upregulated earlier than those in evergreen irises during CA, whereas during DA, the reverse was true. For example, the expression of *jasmonate-zim-domain protein 2* (*JAZ2*) was upregulated on 27 November 2021 in the deciduous iris, but upregulated in the evergreen iris on 11 December 2021. However, the changes in GA signaling genes (positive regulation) were contrasting in both species. For instance, *GA-insensitive dwarf1C* (*GID1C*, a GA-soluble receptor) in the evergreen iris exhibited high accumulation after short-term exposure to the cold during CA, whereas *GID1C* exhibited high downregulation in the deciduous iris. In addition, we noted that *gibberellic-acid-insensitive* (*GAI*) encoding DELLA protein, a key growth repressor in plants, was upregulated on 5 February 2021 (when CA was completed) in the evergreen iris. However, the expression of *GAI* in the deciduous iris reached the maximum level on the first sampling date.

Several transcription factors have been found to regulate the expression of multiple genes related to the cold response in plants [48]. Overall, *CBF2*, *inducer of CBF expression1* (*ICE1*) and *COR27* were significantly upregulated after exposure to extremely low temperatures, especially on 5 February 2021 in the evergreen iris, and then maintained relatively high levels during DA. However, the expression of these genes in deciduous irises remained at relatively low levels throughout the experiment.

### 3.7. Changes in MDA, Proline and Soluble Protein Contents and SOD Activity in Leaf Tissues and the Expression of Related Genes

**Figure 7:**
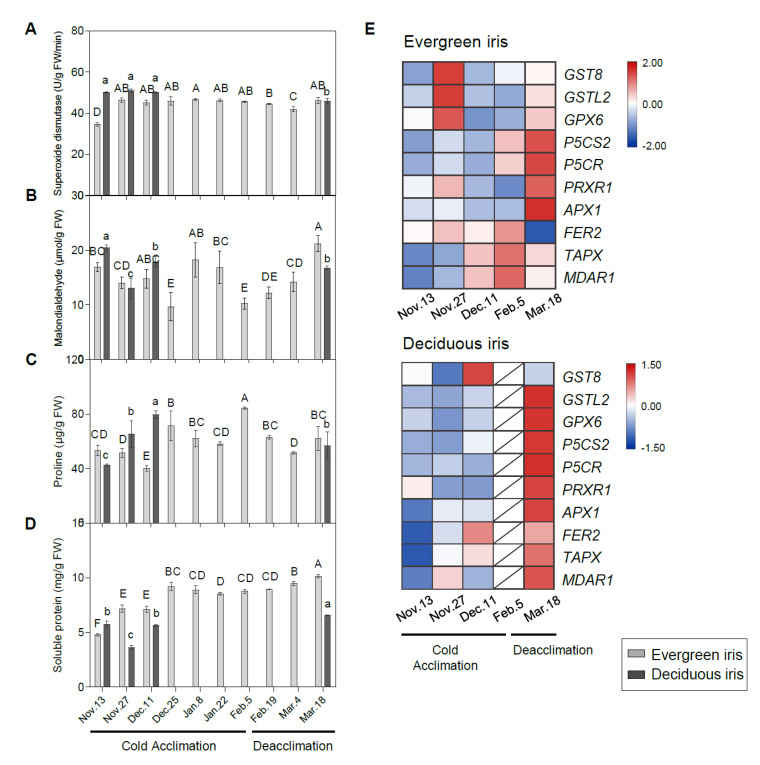
Changes in stress-related indices in the functional leaves of evergreen and deciduous irises and the expression of stress-related genes. Changes in the activities of (**A**) superoxide dismutase, (**B**) malondialdehyde, (**C**) proline and (**D**) soluble protein in the leaves of both species under natural cold acclimation and deacclimation. Cold acclimation process, sampling from 13 November to 5 February 2021; deacclimation process, sampling from 19 February to 18 March 2021. For the deciduous iris, no functional leaves could be sampled from 25 December to 4 March 2021. The data are the means of three biological replicates, with different letters indicating significant differences between treatments according to Duncan’s multiple range test at *p* < 0.05. The push pin represents the standard error. Capital and lowercase letters represent significant differences for relevant parameters within evergreen and deciduous irises, respectively (*p* < 0.05). (**E**) Expression levels of stress-related genes in evergreen and deciduous irises. Gene names are presented in Table S2.

The SOD activity of the evergreen iris substantially increased by 34.46% during early CA from 13 to 27 November 2021, and subsequently remained at relatively high levels until the end of the experiment (Figure 7A). In comparison, the SOD activity of the deciduous iris was maintained at a relatively high level during overwintering. Moreover, the MDA content of both species fluctuated with changes in air temperature during CA and deacclimation (Figure 7B). Notably, MDA content in the evergreen iris increased significantly in January 2021 and showed a relatively low value on 5 February 2021 (when CA was completed), whereas it constantly increased during deacclimation. Furthermore, the MDA content was significantly positively correlated with the LT_50_ value in the deciduous iris, indicating that cold stress aggravated the degree of cell damage (Table 1). 

Overall, the proline content in the evergreen iris presented an increasing trend with temperature dropping during CA and subsequently decreased during deacclimation (Figure 7C). Likewise, the trend of proline in the deciduous iris was similar to that in the evergreen iris. Moreover, LT_50_ and proline showed a high correlation in the evergreen iris (Table 1). The content of SP in the evergreen iris was maintained at a relatively high level during CA and deacclimation, and peaked on 18 March 2021 (Figure 7D). Even though the SP content in the deciduous iris fluctuated, it also reached the maximum value on 18 March 2021.

The expression levels of stress-response genes were measured to provide insight into the stress responses of iris leaves during overwintering (Figure 7E). In summary, most stress-response genes of the deciduous iris were prominently upregulated on 18 March 2021 during deacclimation (Figure 7F); however, they were remarkably upregulated in evergreen irises both on 18 March 2021 and during CA. Two proline biosynthetic genes, *delta1-pyrroline-5-carboxylate synthase 2* (*P5CS2*) and *delta 1-pyrroline-5-carboxylate reductase* (*P5CR*), were extremely highly expressed on 18 March 2021 in both species. 

## 4. Discussion

CA facilitates plants in attaining freezing tolerance and adapting to low temperatures during the winter. During this process, various regulatory mechanisms and metabolic events are activated, which are essential for plants to resist cold. Global warming is leading to range shifts toward the poles at an average rate of 6.1 km per decade and the advancement of spring events [49], which will have a strong impact on plants. The two iris species used in this study have close genetic relationships, consistent habitat environments and similar leaf structures, but they have different overwintering phenotypes (evergreen and deciduous), which interested us in the CA of these two irises species. This study was mainly concerned with the different mechanisms of CA in evergreen and deciduous irises during overwintering. In the context of the lack of appropriate mutants, performing comparisons between closely related species has been proven feasible in numerous studies [50,51,52]. Moreover, in order to avoid the interference of interspecific differences, we compared changing trends rather than values of measured indicators in evergreen and deciduous species. Thus, a comparative study between a pair of closely related irises was conduct in this study, which is very helpful to extend our understanding of CA regulatory mechanisms and discover key pathways and genes of CA in herbaceous perennials.

### 4.1. Different Cold Acclimation Characteristics in Evergreen and Deciduous Irises

Leaves of the evergreen irises experienced a more complete CA process to obtain higher freezing tolerance than the deciduous irises (Figure 1B). However, a short period of unseasonal warm spells from late December to early January 2021 caused evergreen irises to temporarily diminish LFT. Afterward, the LFT of the evergreen iris rapidly increased under extremely low temperatures and reached its maximum in early February 2021. Likewise, the cold-hardy *Rosa rugosa* cultivar could not develop its maximum hardiness after a relatively short period of low-temperature treatment (2 weeks) [53]. The evergreen iris finally lost LFT during deacclimation. These results verified that the acquisition of LFT by CA is reversible, and that unremitting low temperatures are required for CA in herbaceous plants [13]. In contrast, the LFT of deciduous irises was slightly enhanced before leaf senescence (before 11 December 2021) during CA (Figure 1B). Leaf senescence is another strategy that plants evolved in response to cold [54,55]. It is known as an altruistic process, which is critically conducive to the fitness of whole plants by transferring nutrients to other tissues or organs of the plant [56]. A cold-hardiness study of the bark and xylem tissues in deciduous and evergreen peaches (*Prunus persica* L. Batsch) found that the maximum hardiness level attained in deciduous trees was more than twofold greater than that in evergreens [57]. The leaves of the deciduous iris underwent a short period of CA and ultimately died, similar to the deciduous peach, which provided the deciduous iris with optimal fitness. 

### 4.2. Cold Acclimation Activates Carbohydrate Accumulation in Response to Cold for Both Evergreen and Deciduous Irises

In general, the dynamics of the conversion of starch and soluble sugars are present in plants during overwintering. Significant elevations in soluble carbohydrate concentrations and reductions in starch concentrations were exhibited in many perennials during CA, which is widely documented to result in an enhanced freezing tolerance [58,59]. For example, starch converted to soluble sugars during CA was observed in the leaves of *Camellia sinensis*, whereas its starch concentrations increased with decreasing sugar concentrations during deacclimation [13]. However, similar trends were exhibited in the concentrations of soluble sugar and starch in the leaves of evergreen and deciduous irises (Figure 3A–C). Moreover, the concentrations of soluble sugar and starch in evergreen irises reached the maximum levels when CA was completed (on 5 February 2021), thus enhancing their resistance to extremely low temperatures. The concentrations of soluble sugar and starch in the deciduous iris peaked before defoliation, which was consistent with the LFT. Collectively, these results suggested that CA activates carbohydrate accumulation in response to cold in both evergreen and deciduous irises.

The accumulation of starch in leaves after cold treatment is usually neglected, although it has been found in many herbaceous plants [60,61]. Elevated starch concentrations in leaves may be the result of plants’ protective responses. This phenomenon has been studied in *Arabidopsis thaliana* and clonal plantlets of *Broussonetia papyrifera*, which might be a special strategy for herbaceous plants to cope with cold stress [62,63]. Interestingly, starch concentrations in both iris species peaked at their independent ends of CA (Figure 3C), certifying that CA played a persistent role in activating starch accumulation. Moreover, many carbohydrate reserves are necessary at the beginning of overwintering [64], which might be the reason for the accumulation of starch in the deciduous iris before defoliation.

Cold facilitates starch degradation into sugars, which act as important osmoprotectants and play multiple roles in protecting cell membranes from freezing injury [65]. Several *BAM* isoforms have been identified as cold-shock-induced amylase genes [66]. In *Arabidopsis*, *BAM1* and *BAM3* knockout mutants exhibited reduced sugar accumulation during CA [67]. *PtrBAM1* from *Poncirus trifoliata* functions under cold stress through direct regulation by CBF [12]. In the present study, *BAM* was slightly upregulated on 27 November 2021 in both species during early CA; then, a decrease to the lowest temperature induced a pronounced upregulation of *BAM1/3* in the evergreen iris on 5 February 2021 (Figure 3E). Additionally, starch catabolism genes were upregulated much earlier in evergreen irises than in deciduous irises, indicating that the evergreen irises were probably more sensitive to low-temperature stress. Furthermore, changes in TSS concentration did not completely correlate with alterations in the expression of starch catabolism genes in the deciduous iris (on 11 December 2021 during early CA), which might be because metabolite synthesis lags behind gene expression [68]. Overall, these results suggested alterations in the expression of starch catabolism genes, especially *BAM1/3*, indicating differences in starch remobilization and sugar metabolism between the two iris species.

### 4.3. Cold Acclimation Integrates Phytohormone Signaling to Withstand Low Temperature

Auxin plays a central role in endogenous phytohormonal signaling networks, which modulate various life activities and is an integral part of the stress-response mechanism [69]. Under cold stress, decreased IAA levels were observed in *Arabidopsis*, *Davidia involucratei* [70]. Additionally, auxin levels also decline with leaf senescence [71], which may be the reason for the decreased IAA levels in the deciduous iris. However, IAA levels were stable in *Triticum aestivum* ‘Nadro’ (spring wheat), but pronouncedly increased in *Triticum aestivum* var. Mv Emese (winter wheat) and rice after cold exposure [72]. Similarly, the IAA level markedly increased in the evergreen iris during CA, which constantly grew during overwintering (Figure 1C). These results indicated that IAA coordinated plant growth and freezing tolerance during CA, presenting variations depending on genetic background and organs. A decrease in IAA levels and an increase in freezing tolerance were observed in rice seedlings overexpressing *OsGH3.2* [17]. *GH3.2* was significantly upregulated during CA in the evergreen iris, but with an increased IAA level, which might have been due to the remarkable elevation of the auxin biosynthetic gene *IAA27* during CA (Figure 5D). These results indicated that the auxin level is a reasonable monitoring target to cope with cold stress for evergreen irises during CA.

In contrast with IAA, ABA inhibits plant growth and promotes leaf senescence during CA. More importantly, ABA acts as a stress signal because abiotic stresses upregulate its synthesis, especially under cold stress [47]. CA significantly increased the biosynthesis of ABA in *Solanum lycopersicum* [15]. In our study, the ABA level was highly correlated with LT_50_ in both species (Table 1), indicating a crucial role for ABA during the CA of irises. Earlier CA and/or higher freezing tolerance was associated with faster or more remarkable alterations in ABA levels in iris plants. Specifically, the advancement of CA activated the alteration of genes involved in the ABA signaling pathway to facilitate improvements in freezing tolerance. For instance, *PYL*, encoding the ABA receptor, is a positive signaling gene in this pathway. Alleviated cold injury and increased freezing tolerance were exhibited in *Populus trichocarpa* overexpressing *PtPYR1*/*5* and rice overexpressing *OsPYL3/9* [73,74]. In our study, a prominent upregulation of *PYL8* was demonstrated in the evergreen iris, but this was downregulated in the deciduous iris during CA (Figure 6). However, the opposite expression patterns of *PP2C5* were observed in two iris species (Figure 6). In addition, ABA functions under low temperature by activating the expression of CBF genes [75]. In the evergreen iris, ABA and low temperatures might synergistically increase the expression of CBF and elevate freezing tolerance. Therefore, the ABA-dependent PYR8–PP2C5–SnRK2–AREB signaling route is likely more efficient in evergreen irises than in deciduous irises during CA.

CA also activates the accumulation of JA, which participates in the stress and defense responses of plants [76]. For example, the JA levels in rice increased under cold stress [77]. Analogously, JA levels increased during CA and decreased during deacclimation in both iris species (Figure 4C). *Coronative*-*insensitive 1* (*COI1*) and *MYC2* are key positive regulators in the JA signaling pathway [78]. The expression of *CBF/DREB1* and their target genes was reduced in *coi1-1* mutants of *Arabidopsis* under cold treatment [18]. In our study, *COI1* was upregulated during CA in the evergreen iris with the upregulation of *CBF2*, *ICE1* and *COR27*, whereas changes in the expression of these genes were not found in the deciduous iris. Moreover, *MYC2* was upregulated in the evergreen iris during CA; however, it was only upregulated on the first sampling date in the deciduous iris (Figure 6). These results indicated that genes in the JA signaling pathway might prominently function in the evergreen iris during CA, although promote leaf senescence in the deciduous iris [76]. 

In contrast with JA, cold stress accelerates the degradation of GA and leads to growth restrictions [18]. The GA pathway interacts with JA signaling and CBF-dependent CA [79]. In this pathway, the DELLA protein is encoded by *GAI*-mediated growth restriction under cold stress. In *Pyrus ussuriensis*, *GAI* was rapidly upregulated after 12 h cold treatment [80]. Similarly, *GAI* levels in the deciduous and evergreen irises were upregulated on 13 November and 5 February 2021 during CA, respectively. However, the GA_3_ level accumulated, whereas growth was restricted in both iris species during CA (Figure 4D), which suggested that the effect of GA_3_ on plant growth seems to be complicated under cold stress.

### 4.4. Genes Belonging to the Antioxidant System Were Mainly Induced during Deacclimation

During CA, ROS-scavenging enzymes can alleviate oxidative stress and improve freezing tolerance [81]. Cold stress significantly induces antioxidant enzymes and antioxidants in the leaves of tea, *Capsicum annuum* and wheat [21,82,83]. In the present study, genes associated with ROS scavenging, including *glutathione transferase lambda 2* (*GSTL2*), *glutathione peroxidase 6* (*GPX6*), *thylakoidal ascorbate peroxidase* (*TAPX*) and *monodehydroascorbate reductase 1* (*MDAR1*), were upregulated during CA in evergreen irises rather than deciduous irises (Figure 7F), which doubtlessly contributed to the improvements in LFT of evergreen irises. However, most of these genes were significantly upregulated on 18 March 2021 in both species during deacclimation, which might because of the stronger life of plants at that time. Furthermore, cold induces an increase in proline, which acts as a cellular osmotic regulator or radical scavenger to cope with the damage caused by abrupt chilling [84]. Proline accumulates in *Medicago sativa* and wild tomato under cold stress [85]. A prominent increase in proline was also observed in both iris species during CA (Figure 7C). Likewise, the proline biosynthetic genes *P5CS2* and *P5CR* were significantly upregulated on 5 February 2021 in both iris species. In summary, these changes are probably because new leaves of irises could rapidly activate genes involved in the antioxidant system in early spring, although changes in physiological parameters lagged behind gene expression.

### 4.5. The ICE1–CBF–COR Transcriptional Cascade Integrates Multiple Stress Response Pathways during Cold Acclimation

During CA, the ICE1–CBF–COR transcriptional cascade protects plant cells from cold-induced injury and improves freezing tolerance [2]. *ICE1* encodes an MYC-type bHLH transcription factor that can bind to the CBF promoter, thereby activating the expression of *CBF* and target gene *CORs* [86]. The overexpression of *ZjICE1* (isolated from *Zoysia japonica*) in *Arabidopsis* exhibited stronger freezing tolerance and intensely induced the expression of *CBF3* [87]. Cold-activated *CBFs* combine with CRT/DRE cis-elements in the promoters of *COR* genes to stimulate their expression [88], and the overexpression of *CBF* in *Arabidopsis* improves freezing tolerance [10]. In our study, the expressions of *ICE1*, *CBF2* and *COR27* exhibited prominent elevations during CA in the evergreen iris, but were barely transcribed in the deciduous iris (Figure 6).

**Figure 8:**
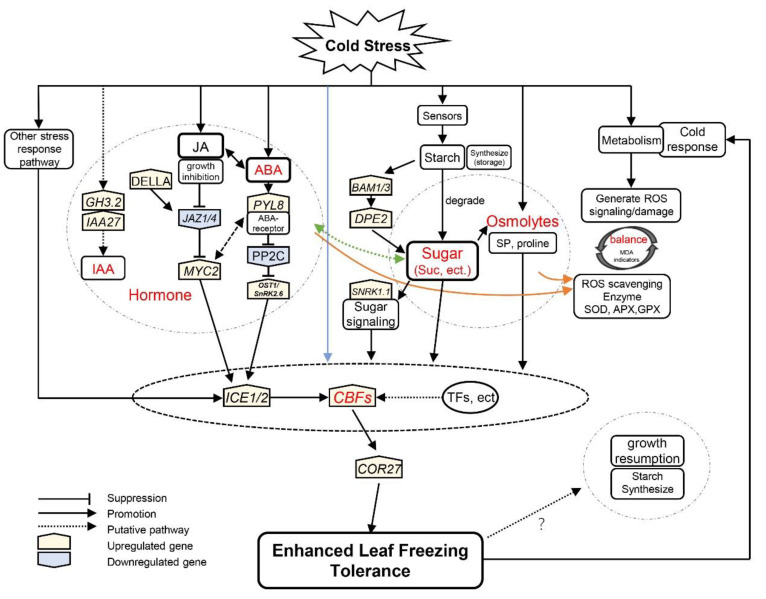
A hypothetical model for iris plants in response to cold stress during winter. Arrows indicate promotion, whereas lines ending with a bar suggest suppression. The blue line indicates that cold signaling partially directly regulates the leaf freezing tolerance of plants through the ICE–CBF–COR cascade. The orange lines indicate phytohormones and sugars that facilitate reactive oxygen species scavenging. The levels of jasmonic acid (JA) were proportional to sucrose concentration in evergreen irises, and the green line indicates a putative pathway. Gene names are shown in Table S2.

The ICE1–CBF–COR transcriptional cascade overlapped with phytohormone signaling and integrated other stress response pathways to respond to cold stress, which is pivotal in improving freezing tolerance [8] (Figure 8). On the one hand, the ICE1–CBF–COR pathway could enhance the degradation of starch to withstand cold stress [13]. For example, *PtrBAM1* from poplar is a CBF regulon member that functions in freezing tolerance by modulating soluble sugar levels [12]. In the evergreen iris, similar trends were present in the expression of *ICE1*, *CBF2*, *COR27* and *BAM1/3*, which were upregulated during CA and peaked at the end of CA (Figure 3E and Figure 6).

On the other hand, phytohormones are involved in the ICE1–CBF–COR pathway in response to cold [76]. In *Vitis vinifera*, ABA activates the expression of the *CBF1* gene [75]. The expression of *COR* genes is also closely related to the biosynthesis and signaling of ABA [75]. Moreover, JA regulates the transcription of *ICE1* by the interaction between *JAZ1/4* and *ICE1/2*, thereby affecting the expression of CBF [18]. In addition, *CBF1* has been found to regulate GA deactivation and the accumulation of DELLA protein when exposed to low temperature in *Arabidopsis* [89]. In the evergreen iris, the levels of IAA, ABA and JA accumulated with the upregulation of *ICE1*, CBF2 and COR27 (Figure 4 and Figure 6).

Furthermore, the cold stress response pathways interact with each other during CA. For instance, JA functions downstream of ABA to motivate the CBF pathway in light-quality-mediated freezing tolerance [19]. The levels of ABA and JA accumulated in evergreen and deciduous irises during CA, indicating that ABA and JA acted synergistically to improve freezing tolerance (Figure 4B,C). Phytohormones also affect sink–source relationships under cold stress [90]. The proposed interaction between sugar and ABA signaling existed in the cold-shock response in wheat [77]. Similar changing trends were exhibited in carbohydrate concentrations and phytohormone levels (except for IAA in the deciduous iris) in the two iris species (Figure 3 and Figure 4). In conclusion, the ICE1–CBF–COR transcriptional cascade might integrate carbohydrates and phytohormones to moderate freezing injury in the leaves of the evergreen iris. However, this regulatory mechanism is not obvious in the deciduous iris potential because of incomplete CA.

## 5. Conclusions

Overall, the evergreen irises achieved stronger LFT than the deciduous irises by more complete CA. Fast and strong CA activated carbohydrate accumulation, ROS scavenging and integrated phytohormone signaling, which exhibited a significant promotion in the improvements in LFT. Moreover, the ICE1–CBF–COR transcriptional cascade might integrate those stress response pathways during CA. These findings provided a hypothetical regulatory network of CA and identified some key genes. The unpredictable contemporary climate forces plants to rapidly respond to environmental signals in winter. However, the underlying molecular mechanisms of cold stress responses, especially in those evergreen herbs, are incomplete. Further studies should aim to determine the interactions between different cold stress response pathways and evaluate the roles and transcriptional networks of candidate genes during CA. Furthermore, critical components in the CA process are yet to be identified.

## Figures and Tables

**Table 1:** Correlation analyses of semi-lethal temperature (LT_50_) and morphological, physiological and biochemical indices in evergreen and deciduous irises during natural cold acclimation and deacclimation.

Evergreen Iris	LT_50_	SER	RCC	LW	FW	NFL	TSS	SUC	STA	IAA	ABA	JA	GA_3_	SOD	MDA	PRO	SP
LT_50_	1																
SER	0.68 *	1															
RCC	0.55	0.45	1														
LW	0.60	0.90 **	0.61	1													
FW	0.66 *	0.85 **	0.61	0.93 **	1												
NFL	−0.14	0.28	−0.55	0.20	0.19	1											
TSS	−0.76 *	−0.45	−0.86 **	−0.61	−0.68 *	0.44	1										
SUC	−0.82 **	−0.67 *	−0.82 **	−0.82 **	−0.85 **	0.24	0.94 **	1									
STA	−0.56	−0.29	−0.61	−0.23	−0.17	0.59	0.55	0.44	1								
IAA	−0.68 *	−0.55	−0.88 **	−0.56	−0.69 *	0.36	0.81 **	0.76 *	0.57	1							
ABA	−0.65 *	−0.18	−0.48	−0.31	−0.38	0.21	0.56	0.60	0.25	0.48	1						
JA	−0.43	−0.05	−0.61	−0.08	−0.26	0.75 *	0.68 *	0.52	0.51	0.65 *	0.22	1					
GA	−0.33	0.10	−0.57	−0.01	0.09	0.82 **	0.46	0.38	0.56	0.29	0.52	0.50	1				
SOD	−0.30	−0.49	−0.48	−0.43	−0.31	0.18	0.40	0.42	0.40	0.38	−0.24	0.33	0.24	1			
MDA	0.50	0.50	0.21	0.42	0.56	0.11	−0.27	−0.47	0.23	−0.37	−0.44	−0.26	−0.08	−0.09	1		
PRO	−0.65 *	−0.31	−0.43	−0.46	−0.45	0.38	0.70 *	0.75 *	0.28	0.26	0.46	0.52	0.53	0.26	−0.42	1	
SP	−0.14	−0.05	−0.67 *	−0.17	−0.08	0.77 **	0.51	0.42	0.48	0.39	−0.01	0.67 *	0.73 *	0.69 *	−0.03	0.44	1
**Deciduous Iris**	**LT_50_**	**SER**	**RCC**	**LW**	**FW**	**NFL**	**TSS**	**SUC**	**STA**	**IAA**	**ABA**	**JA**	**GA_3_**	**SOD**	**MDA**	**PRO**	**SP**
LT_50_	1																
SER	0.54	1															
RCC	0.58	0.90	1														
LW	0.38	0.23	0.63	1													
FW	0.23	0.90	0.65	−0.18	1												
NFL	0.12	−0.12	0.32	0.93	−0.48	1											
TSS	−0.85	−0.70	−0.52	0.08	−0.61	0.39	1										
SUC	−0.94	−0.43	−0.35	−0.05	−0.24	0.19	0.92	1									
STA	−0.29	−0.96 *	−0.89	−0.29	−0.89	0.02	0.46	0.14	1								
IAA	0.46	0.93	0.69	−0.13	0.97 *	−0.47	−0.79	−0.48	−0.85	1							
ABA	−0.96 *	−0.50	−0.66	−0.62	−0.11	−0.39	0.68	0.81	0.30	−0.33	1						
JA	−0.35	−0.32	−0.69	−0.99 **	0.07	−0.90	−0.07	0.00	0.39	0.04	0.59	1					
GA	−0.61	−0.75	−0.96 *	−0.82	−0.40	−0.56	0.39	0.32	0.74	−0.47	0.75	0.86	1				
SOD	−0.29	−0.73	−0.36	0.49	−0.91	0.77	0.75	0.44	0.62	−0.92	0.08	−0.42	0.10	1			
MDA	0.98 *	0.35	0.46	0.43	0.02	0.23	−0.73	−0.91	−0.11	0.26	−0.96 *	−0.37	−0.54	−0.10	1		
PRO	−0.54	−0.81	−0.98 *	−0.75	−0.51	−0.49	0.38	0.26	0.83	−0.55	0.66	0.81	0.99 *	0.19	−0.44	1	
SP	0.81	0.61	0.39	−0.19	0.55	−0.47	−0.99 **	−0.93	−0.35	0.74	−0.63	0.19	−0.26	−0.74	0.71	−0.25	1

Note: Correlation was analyzed using a Pearson’s two-tailed test. Asterisk (*) represents significant at *p* < 0.05, ** represents significant at *p* < 0.01. SER, shoot elongation rate; RCC, relative chlorophyll content; LW, leaf width; FW, fresh weight; NFL, number of the functional leaves; TSS, total soluble sugar; SUC, sucrose; STA, starch; IAA, indole-3-acetic acid; ABA, abscisic acid; JA, jasmonic acid; GA_3_, Gibberellic acid; SOD, superoxide dismutase; MDA, malondialdehyde; PRO, proline; SP, soluble protein.

## Data Availability

Data are contained within the article and Supplementary Materials.

## Supplementary Materials

The following supporting information can be downloaded at: https://www.mdpi.com/article/10.3390/antiox11050977/s1, Figure S1: Leaf epidermis slices of (A) evergreen and (B) deciduous irises. Table S1: Temperatures set for semi-lethal temperature (LT50) detremination (°C) on each sampling date. Table S2: Genes and corresponding primers used for quantitative real-time PCR. Table S3: Changes of relative chlorophyll content (RCC), leaf width (LW), No. of functional leaves (NFL) and fresh weight (FW) in evergreen and deciduous irises during natural cold acclimation and deacclimation. Table S4: Comparison in characteristics of leaf microstructure between evergreen and deciduous irises.
