# Supplementary material for: Integrative Comparative Assessment of Cold Acclimation in Evergreen and Deciduous Iris Species
Source: Antioxidants (Basel). 2022 May 16;11(5):977. doi: 10.3390/antiox11050977 (PMC9137773; doi:10.3390/antiox11050977)
Supplement: Supplementary file 1 [file antioxidants-11-00977-s001.zip › Supplemental Tables.pdf]

Table S1

Temperatures set for semi-lethal temperature ($LT_{50}$) detremination (°C) on each sampling date.

| Sampling date | Nov.13 | Nov.27 | Dec.11 | Dec.25 | Jan.8 | Jan.22 | Feb.4 | Feb.19 | Mar.5 | Mar.18 |
|---|---|---|---|---|---|---|---|---|---|---|
| Temperature 1 | 4 | 0 | 0 | -4 | -4 | -4 | -4 | -5 | 0 | 0 |
| Temperature 2 | 0 | -3 | -4 | -8 | -8 | -8 | -8 | -10 | -4 | -4 |
| Temperature 3 | -3 | -6 | -8 | -12 | -12 | -12 | -12 | -15 | -8 | -8 |
| Temperature 4 | -6 | -9 | -12 | -16 | -16 | -16 | -16 | -20 | -12 | -12 |
| Temperature 5 | -9 | -12 | -16 | -20 | -20 | -20 | -20 | -25 | -16 | -16 |
| Temperature 6 | -12 | -15 | -20 | NA | NA | NA | NA | NA | NA | NA |

Note: NA indicated temperature 6 was not set.

Table S2

Genes and corresponding primers used for quantitative real-time PCR.

| Gene name | Gene full name | *Arabidopsis* homolog | Functional description | Forward/reverse primer sequence (5'-3') |
|---|---|---|---|---|
| reference gene | | | | |
| *ACT7* | *Actin7* | AT5G09810 | cell division, cytoskeleton organization | ACATTGTGCTCAGTGGAGGT/ ACTTCCTCTCTGGTGGAGCA |
| Genes associated with carbohydrate metabolism process | | | | |
| *SEX4* | *starch-excess 4* | AT3G52180 | starch catabolic process | GTAAACGGCCATGCTTTCCG/ TTCTCTGCCCCCATCCAATG |
| *MEX1* | *maltose escess 1* | AT5G17520 | Starch metabolism process | CCCGAATAGCATTTTGCCCG/ ACAAGTCTCGCTCCTTGCTC |
| *SUT4* | *sucrose transporter 4* | AT1G09960 | sucrose metabolic process, sucrose transport | TGCTTGGCTTTGCTACTGGA/ GGGGACTTCTTGGACTGACG |
| *AMY3* | *alpha-amylase-like 3* | AT1G69830 | starch catabolic process | GTGGGCGAGTATTGGGACTC/ ACGTCAAAGGCACCACAAGT |
| *ISA3* | *isoamylase 3* | AT4G09020 | starch catabolic process | TGCGTTCTCGGCAAATGAAG/ TTGTTTCCGTTACGCGTGTG |
| *DPE2* | *disproportionating enzyme 2* | AT2G40840 | starch catabolic process | GGAGAGCCCGTCTTACACAG/ TCCCAACCAGACCCGTAAGA |
| *GLT1* | *glucose transporter 1* | AT5G16150 | response to trehalose | ATGTTGCAGCTAGTGCCCTT/ TGAAGCAGCCATTCCAGTGA |
| *SUS4* | *sucrose synthase 4* | AT3G43190 | sucrose metabolic process | GCGAAATGGCGAGCTGTATC/ TAGGCCGTCGCAAATGTAGG |
| *GWD3* | *phosphoglucan water dikinase* | AT5G26570 | Starch metabolism process | CTACAGAGGCCATGCTTGCT/ TCGGTCAAGCTTCCTGCATT |
| *LSF1* | *Like SEX4 1* | AT3G01510 | starch catabolic process | AACAGCACAAGGATGGCGTA/ GGAAAGCTCCCATATGCCCA |
| *SS3* | *starch synthase 3* | AT1G11720 | Starch biosynthetic process | TGCTCCTGTTGCGTGGTTAT/ CACCGTCGTAGCCTTATCGG |
| *BAM3* | *beta-amylase 3* | AT4G17090 | maltose biosynthetic process, response to cold | TGGCGAGTTCCAGTGCTATG/ TCCCGTAGTCCGAGTTCCAT |
| *GBSS* | *granule bound starch synthase* | AT1G32900 | starch biosynthetic process | ACCGAGTCTTGACAGTGAGC/ TCGTTTGCTGGATCCCACTC |
| *BAM1* | *beta-amylase 1* | AT5G65700 | starch catabolic process | TTCCATTGCATTTGCGGTCG/ TTTGTAGACGATTCCGGCCC |
| *SNRK1.1* | *snf1-related protein kinase 1.1* | AT3G01090 | sugar mediated signaling pathway, abscisic acid-activated signaling pathway | GTTCCACTGGGCATCCTGAA/ TCCTCCCTCGTCTTGTAAAGC |
| Genes associated with phytohormone signaling pathway | | | | |
| *TIR1* | *transport inhibitor response 1* | AT3G62980 | response to auxin | CGCTTCCGGCTCTGTATCAT/ GTCAGCAGAGGCCCCAATAG |

| | | | | |
|---|---|---|---|---|
| *AMT1* | *a-methyl tryptophan resistant 1* | AT5G05730 | auxin biosynthetic process | TGCCGACCCATTTGAAGTGT/ AGAATCTCAGGGCTTGACGC |
| *IAA27* | *indole-3-acetic acid inducible 27* | AT4G29080 | response to auxin | CCTCGGCACTTGAGAAGATGT/ CCAGGGTACGTCACCAACAA |
| *GH3.2* | *auxin upregulated 3* | AT4G37390 | auxin homeostasis, response to auxin | TCTGCCGTTCTATCCGCTTC/ GGTGACGGAATCTCGGACAG |
| *ABA1* | *ABA deficient 1* | AT5G67030 | abscisic acid biosynthetic process, sugar mediated signaling pathway | GGAAAGCTCTGAGACAGGGG/ GGAAAGCTCTGAGACAGGGG |
| *ABA2* | *ABA deficient 2* | AT1G52340 | abscisic acid biosynthetic process, proline biosynthetic process | AGATTCGGGATTCGCGTCAA/ CCTCGAACTCCTCGTTCGTC |
| *AAO3* | *abscisic aldehyde oxidase 3* | AT2G27150 | abscisic acid biosynthetic process | TCCCCTGCGTTCAAGAAGTC/ GGCTCACCAACCGGAAAGTA |
| *NCED3* | *nine-cis-epoxycarotenoid dioxygenase 3* | AT3G14440 | abscisic acid biosynthetic process | GCCGGGCTCATCTACTTCAA/ CTGCCGTCGAAATCGTACCT |
| *PUB44* | *Arabidopsis thaliana plant u-box 44* | AT1G20780 | negative regulation of abscisic acid biosynthetic process | TTCCACCGCTCATGAAGGAC/ AAGTCAGCACCCGAAGACAC |
| *PYL8* | *PYR1-LIKE 8* | AT5G53160 | positive regulation of abscisic acid-activated signaling pathway | AGACCAGGGACGCTAGTGAT/ TCTATGGCTAGACGCTCGGA |
| *PP2C5* | *phosphatase 2C5* | AT2G40180 | abscisic acid-activated signaling pathway | AGGTGGGAAAGAAGCTGCAA/ TGCTGCCTCTAGGGATGACA |
| *SnRK2.4* | *snf1-related protein kinase 2.4* | AT1G10940 | abscisic acid signaling pathway | GCCGAGGTTGAAGATTTGCG/ CCAGCTTGCCGTCGTATTCT |
| *ABF1* | *abscisic acid responsive element-binding factor 1* | AT1G49720 | abscisic acid-activated signaling pathway | AAGGCCTACCAGCTGAGTCT/ CGCAATCCTCTTCCTCCGTT |
| *ABF4* | *abscisic acid responsive element-binding factor 4* | AT3G19290 | abscisic acid-activated signaling pathway | TGGTGAGCAGTGGATTGGTG/ AGGTACTGGTGACAACGACG |
| *AREB3* | *ABA-responsive element binding protein 3* | AT3G56850 | abscisic acid-activated signaling pathway | TCGTGCGGTTCCCCAAATAA/ ATCTTATCCGTGTCCCCCGA |
| *SnRK2.6* | *snf1-related protein kinase 2.6* | AT4G33950 | abscisic acid-activated signaling pathway | AATAGCGGAGGCCACAATCC/ TGGCATTGACTATCTCGCCG |
| *HOS1* | *high expression of osmotically responsive genes 1* | AT2G39810 | response to cold | TTCTACGATGCATTGGGCGT/ AACCGGCCACTTTCAATCCT |
| *ICE1* | *inducer of CBF Expression 1* | AT3G26744 | abscisic acid-activated signaling pathway; response to cold, freezing | CGACATCCTTACCAAGCCCA/ CCATCAAGTGCCCTCATCGT |
| *COI1* | *coronative insensitive 1* | AT2G39940 | jasmonic acid mediated signaling pathway | TTGGAAGAGTTTGGTGGGGG/ ACAGGCATCTCGTTTGTCCC |
| *JAZ2* | *jasmonate-zim-domain protein 2* | AT1G74950 | response to jasmonic acid | AGCCAACCCAAGTCACTGTT/ TATGGTGCCCTGGCATTGAT |
| *MYC2* | *jasmonate insensitive 1* | AT1G32640 | Phytohormone（JA）signaling | TCGATTCGGAGAACGAGAGC/ CGCTTCCGTGCCCATTATTC |
| *CBF2* | *C-repeat binding factor 2* | AT4G25470 | cold acclimation; response to cold; regulation of transcription | TTGGGAGAGAATTCGCGGG/ ATCCCCTCCGCCATATCCTT |
| *CBF4* | *C-repeat binding factor 4* | AT5G51990 | regulation of transcription | GCCGTCTAGTTCGTCGGTAG/ CGTCTCCCCTGCATCATAGC |
| *COR27* | *cold regulated gene 27* | AT5G42900 | response to cold | TGGTTGCATGACAGAGGCAT/ CCACAGACGGAACCACTTGA |
| *GA20ox2* | *gibberellin 20-oxidase* | AT5G51810 | gibberellin biosynthetic process, response to gibberellin | TCATTCAAACCCTCGGCGAA/ GAGTCGTTGCCCTCGAAGAA |
| *GID1c* | *GA insensitive dwarf1C* | AT5G27320 | gibberellin mediated signaling pathway | TCCGCTTACGATGACGGATG/ ACATTATGCGCGATGTTGCC |
| *GAI* | *gibberellic acid insensitive* | AT1G14920 | negative regulation of seed germination, jasmonic acid mediated signaling pathway, | AGCAGGACCAGTTGATGTCG/ CATGCTCGCCTGCTTGAAC |

| | | | | |
|---|---|---|---|---|
| *SPY* | *spindly* | AT3G11540 | gibberellic acid mediated signaling pathway, regulation of reactive oxygen species metabolic process | AGGGATGATGGCTTGCAGAC/ CAGGCAGACGAACCAACTCT |
| Genes associated with stress response process | | | | |
| *GST8* | *glutathione transferase 8* | AT1G78380 | response to oxidative stress | CACCTATGAGACCAACGCCA/ CCTTGTGGGGATCAGAGAGC |
| *GSTL2* | *glutathione transferase lambda 2* | AT3G55040 | response to stress | GTGGATATCGCATACGCTCCA/ GCCGAGTTGATGTGTAGGCA |
| *GPX6* | *glutathione peroxidase 6* | AT4G11600 | response to oxidative stress | TTTCCGTGCAACCAGTTTGG/ GGTGAAGCCTTGTCCCCATT |
| *P5CS2* | *delta1-pyrroline-5-carboxylate synthase 2* | AT3G55610 | L-proline biosynthetic process; | AAGCAACGTAGAGGGAGCAG/ GCATTGGACACAACGAGGTC |
| *P5CR* | *delta 1-pyrroline-5-carboxylate reductase* | AT5G14800 | proline biosynthetic process | TCGAGATCCTCAACGACAGC/ CAGACCACTCCTGCAAGTCT |
| *PRXR1* | *PRXR1* | AT4G21960 | response to oxidative stress | AAGCTGGTGCATCGTCTGTA/ GTCCAGTATCATCGGGGTCC |
| *APX1* | *ascorbate peroxidase 1* | AT1G07890 | oxidation-reduction process | CTACCTGATGCGACCGAAGG/ ATGTCCATGCTCCCTCGAAC |
| *FER2* | *FERRITIN 2* | AT3G11050 | response to abscisic acid, response to oxidative stress | AGCTTGCTGACTTTGTGGAGA/ ATCAAAATGCCAGACCCCGT |
| *TAPX* | *thylakoidal ascorbate peroxidase* | AT1G77490 | cold acclimation, hydrogen peroxide mediated signaling pathway | ATCCTCCAGAGGGTGTCTCC/ TGTTCACACGGTTGGAAAAGC |
| *MDAR1* | *monodehydroascorbate reductase 1* | AT3G52880 | oxidation-reduction process | TGCCGTGGGATTTGATTCTGA/ TTTCCGCCAACTCCTACCAC |

Table S3

Changes of relative chlorophyll content (RCC), leaf width (LW), No. of functional leaves (NFL) and fresh weight (FW) in evergreen and deciduous irises during natural cold acclimation and deacclimation.

| Sampling stage | Deciduous iris | | | | Evergreen iris | | | |
|---|---|---|---|---|---|---|---|---|
| | RCC | LW (mm) | FW (g) | NFL | RCC | LW (mm) | FW (g) | NFL |
| Nov.13 | 47.27±1.74a | 21.20±0.62a | 1.33±0.09b | 6.60±0.55a | 59.70±1.45a | 23.93±0.40b | 1.83±0.06b | 4.80±0.45fg |
| Nov.27 | 36.97±2.39c | 17.20±0.36b | 0.96±0.04b | 5.60±0.55b | 56.63±1.11b | 22.37±0.40bc | 1.56±0.04c | 4.40±0.55g |
| Dec.11 | 31.70±1.61d | 14.73±0.25c | 0.31±0.04c | 4.40±0.55c | 52.33±1.84c | 18.70±6.15d | 1.32±0.09d | 5.00±0.71efg |
| Dec.25 | NA | NA | NA | NA | 48.87±1.19d | 13.27±0.59e | 0.67±0.08e | 5.40±0.55def |
| Jan.8 | NA | NA | NA | NA | 47.13±1.56d | 12.23±0.25e | 0.34±0.06f | 5.20±0.45ef |
| Jan.22 | NA | NA | NA | NA | 39.10±1.25g | 11.87±0.40e | 0.25±0.02fg | 5.60±0.55cde |
| Feb.5 | NA | NA | NA | NA | 43.17±0.91f | 12.80±0.30e | 0.12±0.01g | 6.00±0.71bcd |
| Feb.19 | NA | NA | NA | NA | 46.53±0.97de | 20.20±0.36cd | 0.68±0.06e | 6.20±0.45abc |
| Mar.4 | NA | NA | NA | NA | 44.17±2.21ef | 23.00±0.62bc | 1.65±0.21c | 6.40±0.55ab |
| Mar.18 | 43.50±1.75b | 14.57±0.60c | 3.04±0.46a | 3.40±0.55d | 51.57±1.66c | 27.53±0.93a | 2.98±0.20a | 6.80±0.45a |

Note: Data are means ± (standard error) of ten independent biological replicates with different letters indicating significant differences between treatments according to Duncan's multiple range test at $p$ <0.05. NA indicated no plant material for determination.

Table S4

Comparison in characteristics of leaf microstructure between evergreen and deciduous irises.

| Species | Characteristics of leaf structure | | | | | | |
|---|---|---|---|---|---|---|---|
| | Leaf thickness (um) | Epidermal cell size | | No. of layer of mesophyll cell with chloroplast | Stomatal apparatus size | | Stomatal apparatus densities (entries/mm$^2$) |
| | | Length (um) | Width (um) | | Major axis (um) | Minor axis (um) | |
| Evergreen iris | 622.9 | 27.5 | 22.5 | 7 | 27.2 | 27.2 | 267 |
| Deciduous iris | 585.3 | 12.5 | 11 | 7 | 20 | 16.7 | 286.07 |
