# Supplementary figures and images for: Integrative Comparative Assessment of Cold Acclimation in Evergreen and Deciduous Iris Species
Source: Antioxidants (Basel). 2022 May 16;11(5):977. doi: 10.3390/antiox11050977 (PMC9137773; doi:10.3390/antiox11050977)

A

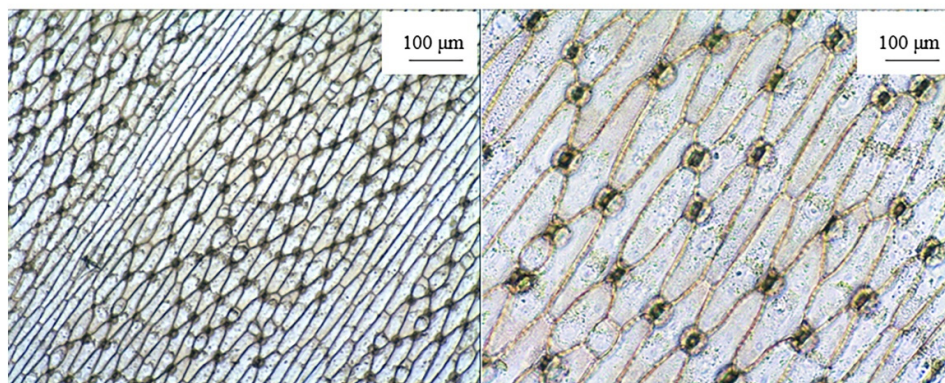

B

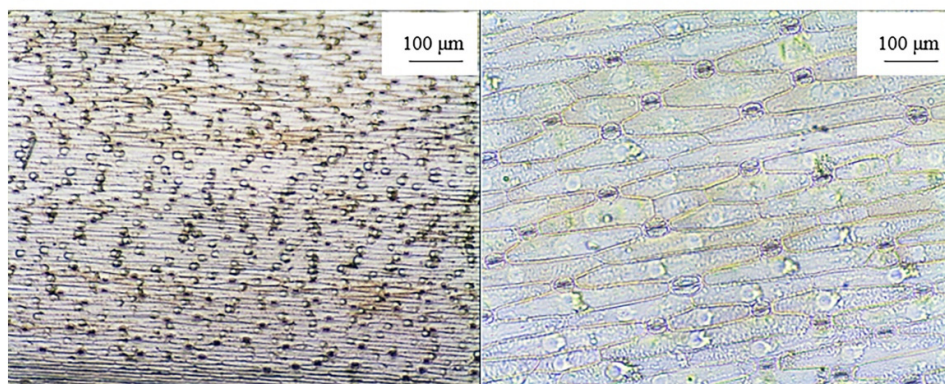

Figure S1. Leaf epidermis slices of (A) evergreen and (B) deciduous irises.

Supplement: Supplementary file 1 [file antioxidants-11-00977-s001.zip › Supplemental figure.pdf]
